# Atrophic Nonunion in Fifth Toe Fractures: A Case Report Linking Occult Infection and Fifth Toe Fracture

**DOI:** 10.7759/cureus.106230

**Published:** 2026-03-31

**Authors:** Chris Lamprecht, Caitlin Curtis Crocker, Miqi Wang

**Affiliations:** 1 Department of Orthopaedic Surgery and Sports Medicine, University of Florida College of Medicine, Gainesville, USA

**Keywords:** fifth toe fracture, nonoperative management, nonunion fracture, occult infection, phalangeal fractures

## Abstract

Phalangeal fractures are the most common fractures in the foot, with the vast majority completely healing with conservative management. The overall nonunion rate for fractures of the lesser phalanges is not well documented in the literature due to their rarity. The following cases explore two unusual presentations of fifth toe closed fracture with delayed union, suggesting a potential link between infection and delayed healing in apparently closed fractures. The first patient, a 46-year-old female with a history of smoking and synphalangism, developed persistent pain and swelling following a toe fracture. Imaging confirmed atrophic nonunion, and cultures from her eventual amputation identified Clostridium species, suggesting occult infection as a contributing factor. The second patient, a 59-year-old female with anemia and synphalangism, presented with delayed healing of a similar fracture. Empirical antibiotic treatment resulted in complete resolution of symptoms and radiographic healing, further supporting a potential infectious etiology. Occult infection may be a contributing factor in cases of delayed union, even for closed toe fractures. Empirical antibiotic therapy may be a viable nonoperative approach in select patients. Further studies are needed to investigate infection as a potential etiology of atrophic nonunion in closed toe fracture healing in order to guide optimal management strategies.

## Introduction

Phalangeal fractures are the most common fractures in the foot, accounting for nearly 10% of fractures treated in primary care, with a predilection for lesser toe fractures [1-3]. These fractures are often caused by direct trauma [1,2,4]. The vast majority of phalangeal fractures, especially those involving the lesser toes, do not result in significant clinical disability, as they typically heal well with conservative management [1-3,5]. While the overall nonunion rate for fractures is approximately 1.9%, specific nonunion rates for toe fractures are not well-documented in the literature due to their rarity [6].

Initial treatment strategies for closed, nondisplaced fractures of the lesser toes include buddy taping to an adjacent digit, combined with the use of a rigid-sole shoe to enable early full weight-bearing and minimize disability [1-4]. These measures are generally employed for about three weeks in children or four to five weeks in adults, which is sufficient for achieving adequate union [2].

Overall, surgical intervention for non-complicated fifth toe fractures is very rare [1-4]. There are no large cohort studies that establish a standard of care or etiologies definitively linking causes for such non-unions. The following cases explore two unusual presentations of fifth toe fracture delayed union, suggesting a potential link between infection and delayed healing.

## Case presentation

Case 1

**Figure 1:**
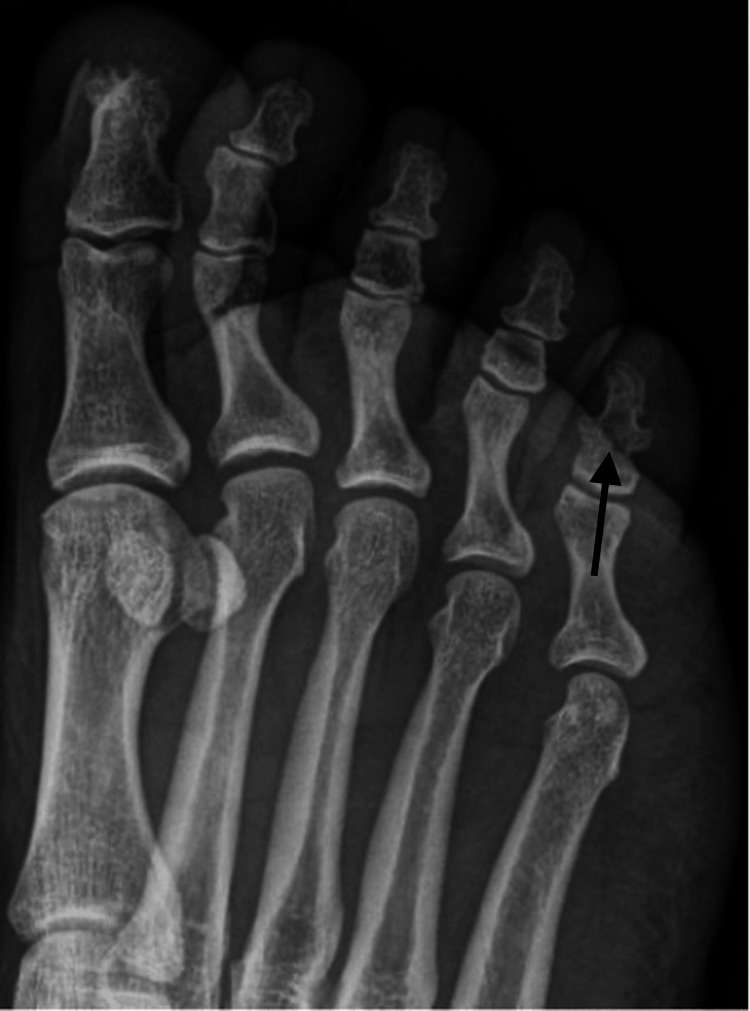
Oblique radiograph of the right foot taken at the time first clinic visit showing a transverse fracture of the distal phalanx of the fifth toe.

The first patient is a 46-year-old female with a history of 15 pack-years of cigarette smoking and synphalangism of the distal and middle phalanges of the right fifth toe. Her medical history is notable for prior corticosteroid exposure in the setting of bilateral hip osteoarthritis and previous left hip osteonecrosis sustained after a fall, for which she underwent surgical intervention approximately two years prior without complications related to infection or delayed healing. The patient originally sustained her injury when she tripped over her dog. X-rays completed at the time of injury displayed a closed fracture of the right fifth distal phalanx without any concerns of nailbed injury (Figure 1). She was initially treated with a postoperative shoe and buddy taping.

**Figure 2:**
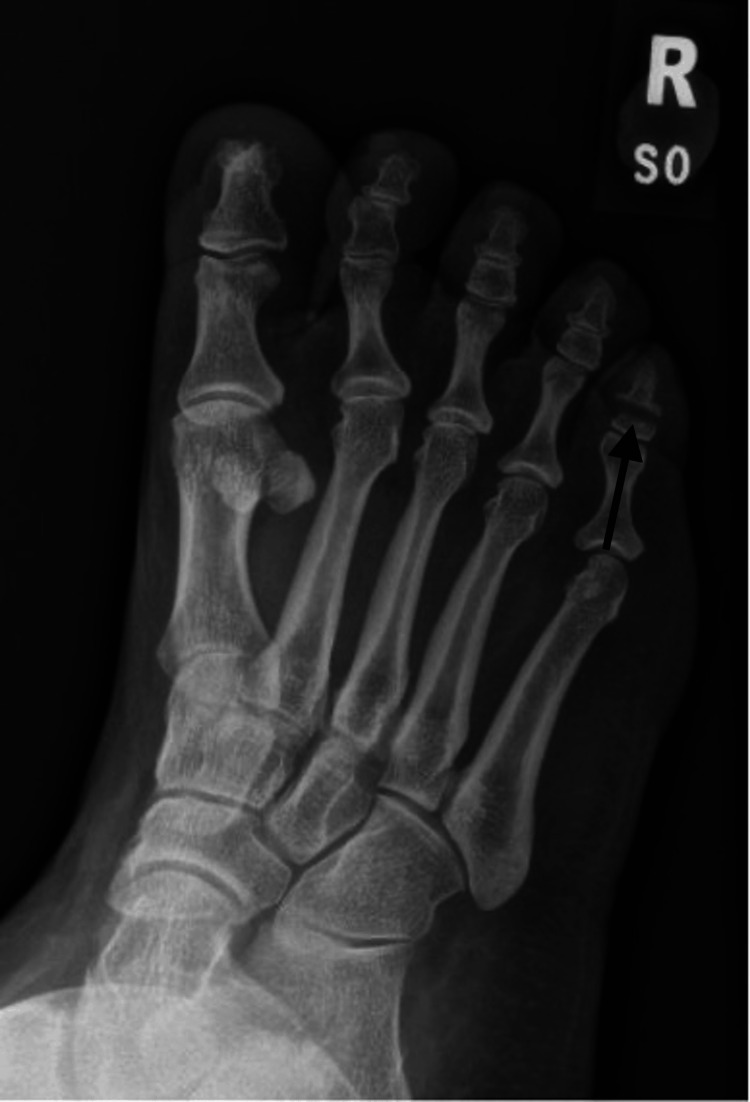
Oblique radiograph of the right foot taken three months post-injury showing a transverse fracture of the distal phalanx of the fifth toe with resorption but no callus formation.

Three months later, she had continual pain, swelling, and discoloration of her fifth toe that was exacerbated with ambulation. Radiographic imaging showed the continued presence of a fracture line with resorption but no callus formation (Figure 2). With the absence of significant healing on follow-up imaging, a conservative treatment approach was initially pursued, including vitamin D, calcium, postoperative rigid shoe, and buddy taping.

**Figure 3:**
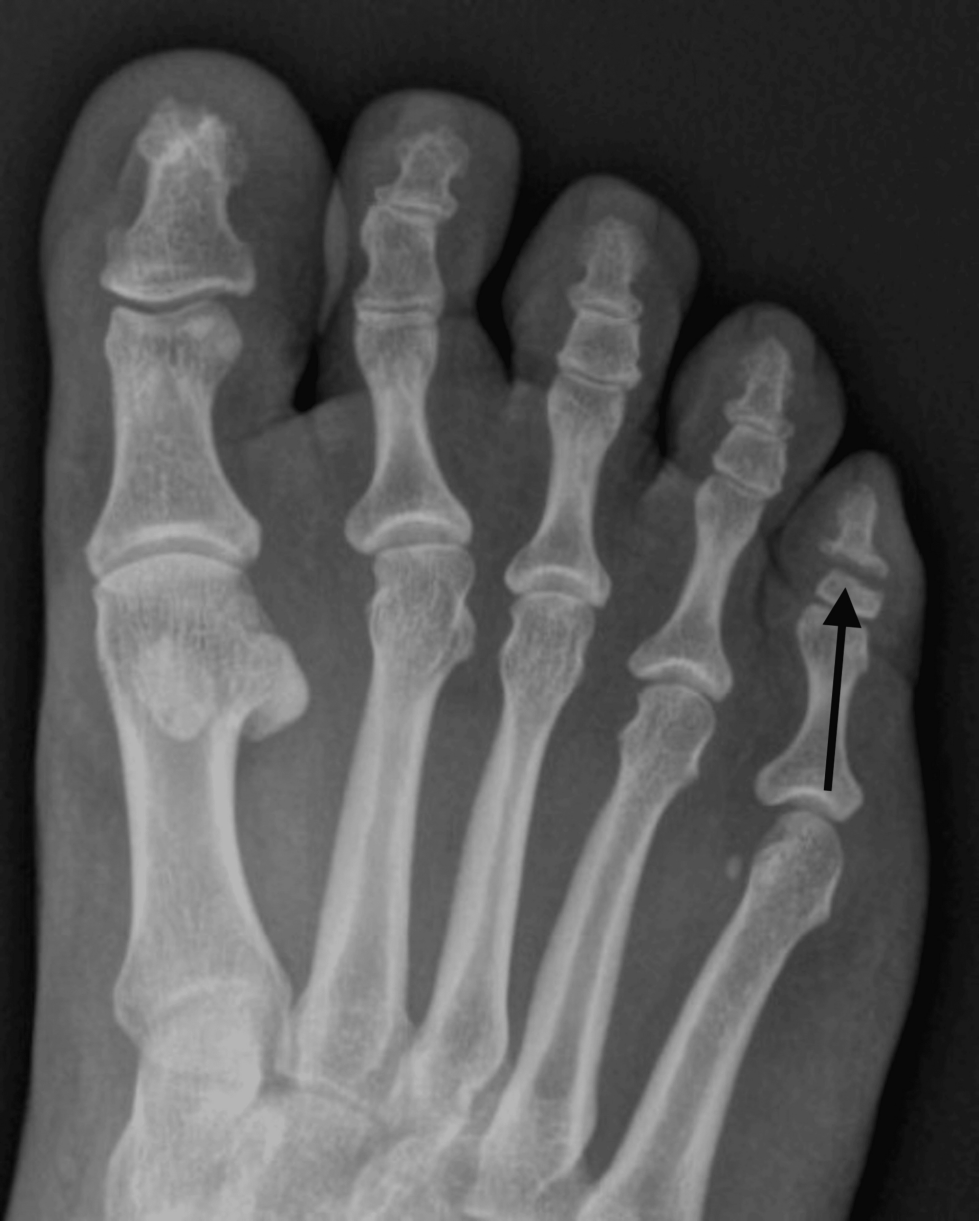
Anterior-posterior radiograph of the right foot taken six months post-injury with persistent nonunion of the fracture.

She returned to the office three months later with continued swelling and pain. Radiographs again demonstrated persistent nonunion with a similar appearance (Figure 3). The patient denied any history of open wounds, fevers, chills, malaise, drainage, or ascending erythema.

Given the persistence of symptoms and radiographic findings of atrophic nonunion, surgical treatment options were explored, including nonunion takedown with open reduction and internal fixation versus amputation. The patient elected to proceed with amputation of the fifth toe. Given the atrophic nature of the fracture, which raised suspicion for infection, a bony sample of the fracture site was sent for culture. Cultures identified non-perfringens Clostridium. Postoperatively, the patient experienced complete pain relief and a rapid return to normal function. At the postoperative one-year follow-up, the patient was doing well and reported no clinical symptoms.

Case 2

**Figure 4:**
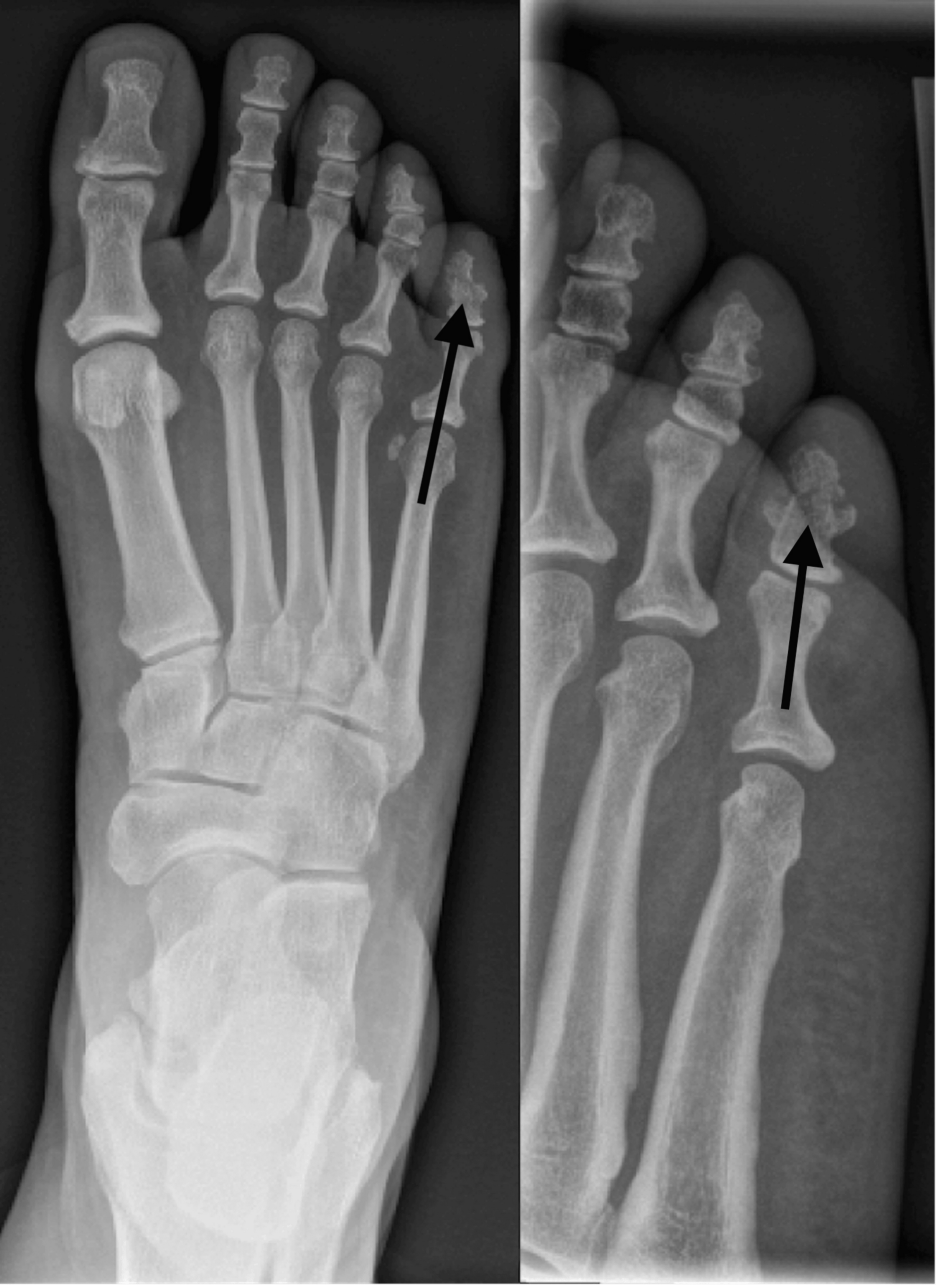
Anterior-posterior and oblique radiographs of the right foot taken at the time of initial injury displaying a nondisplaced fracture of the fifth toe through the distal-middle phalanx.

The second patient is a 59-year-old female with a past medical history notable for distant resolved anemia (hemoglobin 10.9 g/dL corrected with oral iron supplementation nine years prior) and fifth toe synphalangism presented to the orthopedic clinic with right fifth toe pain. Symptoms began after a crush injury to the fifth toe. The patient denied any soft tissue injury or concern for nailbed injury at that time. Initial radiographs revealed a nondisplaced fracture through the diaphysis and tuft of the distal-middle right fifth phalanx (Figure 4). She was treated conservatively with over-the-counter medications and wide-toe box shoes.

**Figure 5:**
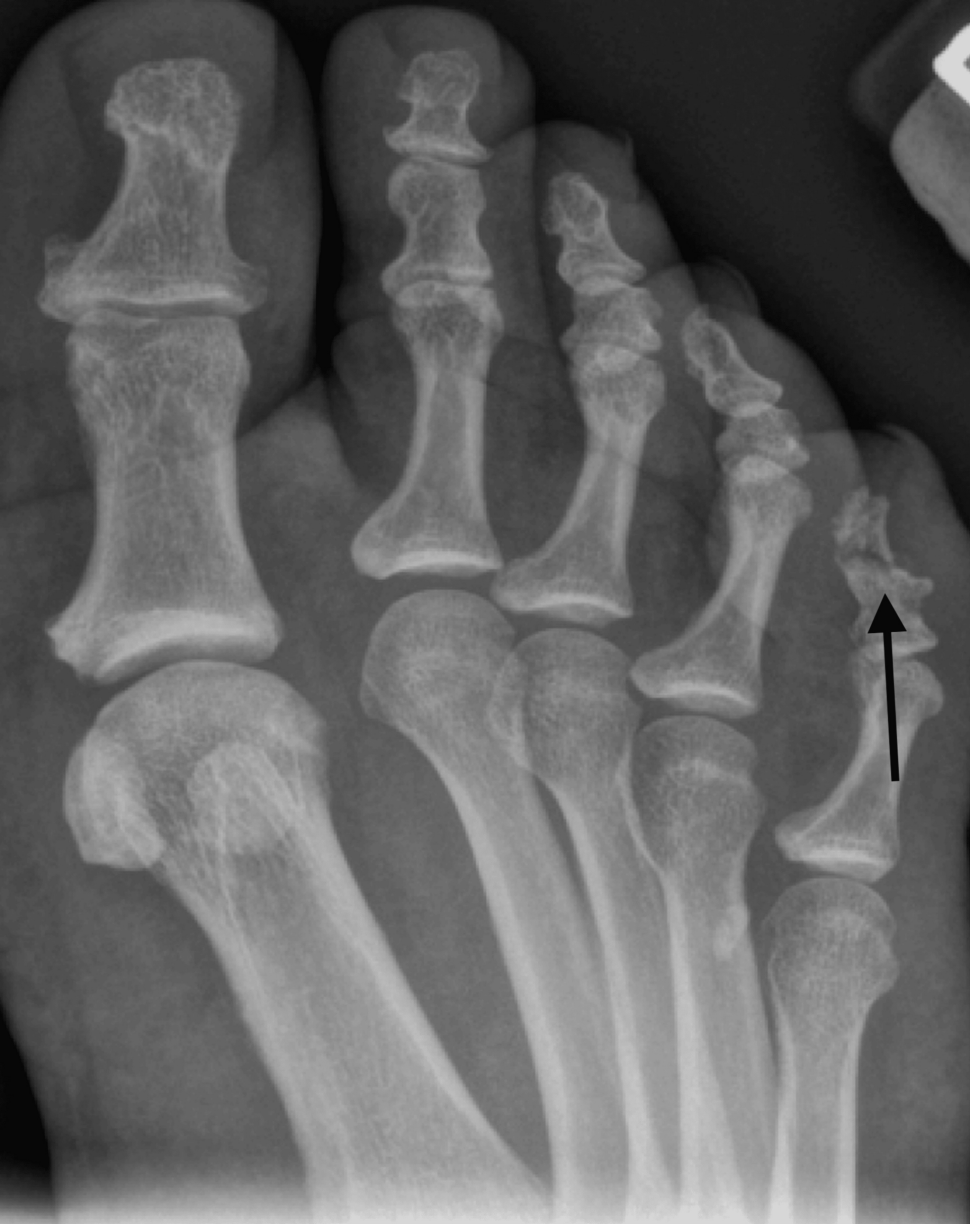
Oblique radiograph of the right foot taken three months post-injury displaying persistent fracture line with resorption and no callus formation of the fifth toe.

At her clinic visit three months following the initial injury, radiographs demonstrated a persistent fracture line with resorption and no callus formation (Figure 5). The patient denied any history of open wounds, fevers, chills, malaise, drainage, or ascending erythema.

After shared decision making with the patient, she opted for empirical treatment for a possible occult infection of the fracture with 10 days of trimethoprim-sulfamethoxazole (TMP-SMX). The patient sustained an adverse reaction to the antibiotic, discontinuing the TMP-SMX following eight days of administration.

**Figure 6:**
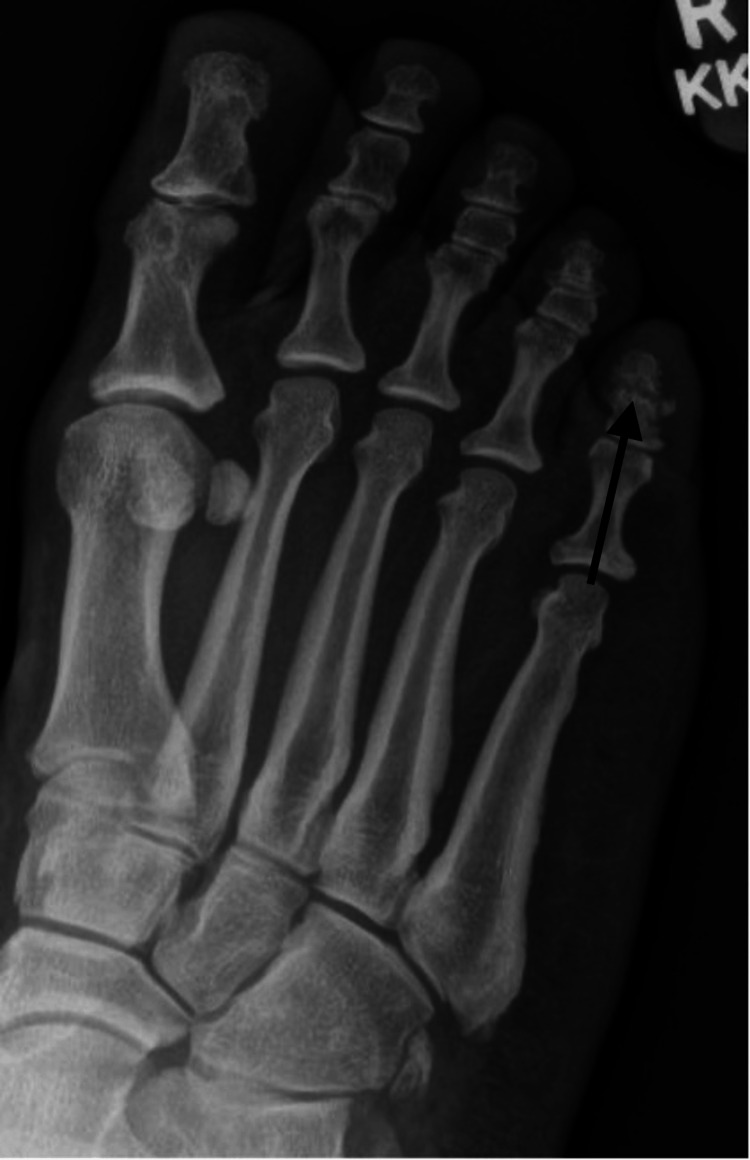
Oblique radiograph of the right foot taken one month post-antibiotic treatment interval healing of the fracture.

**Figure 7:**
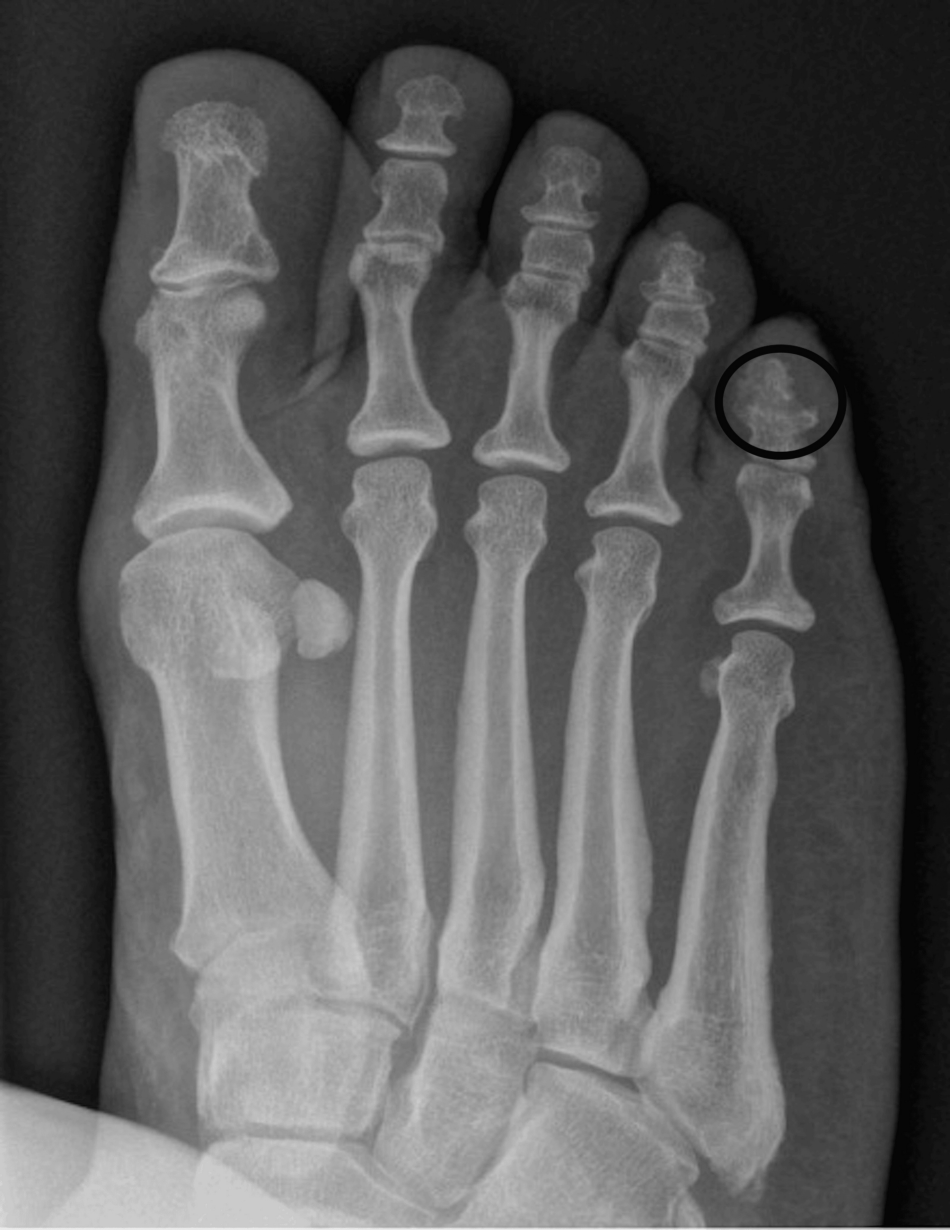
Oblique radiograph of the right foot taken two months post-antibiotic treatment with complete healing of fracture.

One month post-antibiotic treatment, the patient reported significant pain improvement in the fifth toe. Radiographs demonstrated interval healing of the fracture (Figure 6). During her follow-up visit two months post-antibiotic treatment, she had complete resolution of pain and radiographs demonstrated complete healing of the fracture (Figure 7).

## Discussion

Prior reports of painful nonunions in the lesser toes have described cases where surgical stabilization successfully achieved healing, though intraoperative cultures were not obtained [3]. Our cases contribute additional evidence by demonstrating a potential infectious component in closed fracture delayed union. The first patient required surgical intervention, opting for amputation, after conservative management failed. Intraoperative cultures revealed infection with non-perfringens Clostridium. Non-perfringens Clostridium is a rarely reported pathogen involved in toe nonunion. The second patient demonstrated a similarly rare delay in fracture healing. Given the infectious etiology of the first patient, empiric antibiotic therapy was offered. Despite complications from an adverse drug reaction, the second patient experienced appropriate and timely healing of her fracture following treatment. 

Potential confounding factors that may influence fracture healing or infection risk were considered. The first patient had a history of tobacco use and prior corticosteroid exposure, both of which have been associated with impaired healing, though she had no complications related to infection or delayed healing in previous orthopedic procedures. The second patient had no significant comorbidities, with only a remote history of resolved anemia. Neither patient had active systemic risk factors for infection or delayed healing at the time of presentation, such as diabetes mellitus, immunosuppression, or ongoing steroid use. While these factors may contribute to healing variability, they do not fully account for the clinical course observed in these cases. Unlike previous case reports that did not explore infectious causes, our findings provide evidence that bacterial infection may be an underrecognized contributor to delayed healing in unsuspecting closed fifth toe fractures.

Painful nonunions of the lesser toe distal interphalangeal joints have been described in prior literature. Foo and Wee reported a patient with synphalangism of the fourth toe who had a distal phalangeal nonunion. Following failure of conservative treatment, she was successfully treated with interphalangeal joint arthrodesis. During this procedure, the fracture site was debrided of fibrous tissue and fixated through the placement of a headless compression screw. Intraoperative cultures were not taken. The patient achieved union of the fracture [3].

Mansur described another case of a fourth distal phalanx nonunion treated with fibrous excision, bone grafting, and screw fixation. Cultures were not taken during this procedure. Union was achieved in this case by six months post-operatively [4]. 

In both cases, debridement of the fracture site was performed, and union was achieved; however, cultures were not taken. It is possible that infection could have played a role in these fractures, but this is not confirmed. Further studies that utilize intraoperative cultures may identify a bacterial source of nonunion.

The administration of antibiotics for presumed closed, atrophic, delayed union of the fifth toe represents a low-risk intervention with the potential to resolve the condition without surgical intervention, provided there is no adverse drug reaction. We hypothesize that the nail bed could serve as a source of infection even in closed fractures, given its proximity to the distal interphalangeal joint. Based on these clinical observations, further research is warranted to investigate infection as a potential factor contributing to nonunion in toe fractures.

Based on experience from these patients, the senior author has made alterations to practice. We now treat similar-appearing atrophic non-healing fractures of the fifth distal phalanx with a short course of antibiotics. Another patient is currently being followed by the senior author for nonunion and has finished a seven-day course of TMP-SMX. At the six-week follow-up, the patient already had bridging bone formation with pain improvement. At the three-month follow-up, the patient showed complete radiographic union of the fracture site.

This case series is limited by a relatively short follow-up period (one year) and the absence of culture results for the second patient, who was treated empirically for a suspected occult infection. Due to the small cohort size, the conclusions drawn are constrained. Future research should focus on larger-scale prospective studies to investigate the prevalence of infection as an etiological factor in the nonunion of closed fifth toe fractures.

## Conclusions

While existing studies have focused on surgical fixation for symptomatic nonunions, our findings suggest that obtaining intraoperative cultures and considering empiric antibiotic therapy may be valuable additions to treatment protocols. It is uncommon to encounter an infected nonunion in the absence of an associated wound or prior surgical incision, making this a rare clinical scenario with limited cohort-based research available. Future research should investigate the prevalence of infection in closed toe fracture nonunions to better guide management strategies.
